# Structural Studies Reveal that Endosomal Cations Promote Formation of Infectious Coxsackievirus A9 A-Particles, Facilitating RNA and VP4 Release

**DOI:** 10.1128/jvi.01367-22

**Published:** 2022-11-30

**Authors:** Aušra Domanska, Zlatka Plavec, Visa Ruokolainen, Benita Löflund, Varpu Marjomäki, Sarah J. Butcher

**Affiliations:** a Faculty of Biological and Environmental Sciences, Molecular and Integrative Bioscience Research Programme, and Helsinki Institute of Life Sciences-Institute of Biotechnology, University of Helsinki, Helsinki, Finland; b Department of Biological and Environmental Sciences, Nanoscience Center, University of Jyväskylä, Jyväskylä, Finland; Instituto de Biotecnologia, UNAM

**Keywords:** A-particle, AF4, CVA9, VP4, albumin, cryo-EM, endosomal ionic composition, virus structure

## Abstract

Coxsackievirus A9 (CVA9), an enterovirus, is a common cause of pediatric aseptic meningitis and neonatal sepsis. During cell entry, enterovirus capsids undergo conformational changes leading to expansion, formation of large pores, externalization of VP1 N termini, and loss of the lipid factor from VP1. Factors such as receptor binding, heat, and acidic pH can trigger capsid expansion in some enteroviruses. Here, we show that fatty acid-free bovine serum albumin or neutral endosomal ionic conditions can independently prime CVA9 for expansion and genome release. Our results showed that CVA9 treatment with albumin or endosomal ions generated a heterogeneous population of virions, which could be physically separated by asymmetric flow field flow fractionation and computationally by cryo-electron microscopy (cryo-EM) and image processing. We report cryo-EM structures of CVA9 A-particles obtained by albumin or endosomal ion treatment and a control nonexpanded virion to 3.5, 3.3, and 2.9 Å resolution, respectively. Whereas albumin promoted stable expanded virions, the endosomal ionic concentrations induced unstable CVA9 virions which easily disintegrated, losing their genome. Loss of most of the VP4 molecules and exposure of negatively charged amino acid residues in the capsid’s interior after expansion created a repulsive viral RNA-capsid interface, aiding genome release.

**IMPORTANCE** Coxsackievirus A9 (CVA9) is a common cause of meningitis and neonatal sepsis. The triggers and mode of action of RNA release into the cell unusually do not require receptor interaction. Rather, a slow process in the endosome, independent of low pH, is required. Here, we show by biophysical separation, cryogenic electron microscopy, and image reconstruction that albumin and buffers mimicking the endosomal ion composition can separately and together expand and prime CVA9 for uncoating. Furthermore, we show in these expanded particles that VP4 is present at only ~10% of the occupancy found in the virion, VP1 is externalized, and the genome is repelled by the negatively charged, repulsive inner surface of the capsid that occurs due to the expansion. Thus, we can now link observations from cell biology of infection with the physical processes that occur in the capsid to promote genome uncoating.

## INTRODUCTION

Coxsackievirus A9 (CVA9) belongs to the *Enterovirus* genus, species *Enterovirus B* (EV-B) in the family *Picornaviridae*. CVA9 can cause pediatric aseptic meningitis and neonatal sepsis (1–4). Like other picornaviruses, CVA9 has a small, ~30-nm-diameter, nonenveloped viral capsid. Its positive-sense single-stranded RNA genome is ~7,500 bases long and encodes 4 structural proteins (VP1 to 4) and 7 nonstructural proteins (2A to C and 3A to D) (5). Expanded A-particles have been described for many enteroviruses, occurring at early steps during virus entry (6–10). It is thought that VP4 must be present in the A-particle to be infectious, but VP4 is very difficult to detect (11). Many picornaviruses, including members of the EV-B species, accommodate a lipid factor in the VP1 hydrophobic pocket. Palmitate is a commonly found lipid factor or fatty acid in enteroviruses (12). Expulsion of the lipid factor is associated with virion expansion. Once expansion has occurred and the capsid has been endocytosed, genome release can occur (13). It has been shown that receptor binding can trigger a loss of the lipid factor and virion expansion in some EV-B members (14), but not in CVA9 or echovirus 1 (E1) (15–17). In addition, CVA9 and E1 do not depend on low pH for genome release, unlike members of the *Enterovirus A* species, indicating that these viruses use additional cues for uncoating (15, 18). Despite extensive research, the physiological triggers leading to enterovirus uncoating after internalization, other than receptor binding and low pH in the endosomes, are poorly understood (19). Some studies have reported serum albumin as an uncoating cue for E1, E12, and coxsackievirus B3 (6, 20, 21).

It is well established that acidification is key to endosome maturation. In addition to proton influx during endosome maturation, the concentrations of other ions in the endosomal lumen, such as sodium, potassium, and calcium, also change over time due to ion channels and pumps present in the endosomal membranes (22, 23). The absolute values of ion concentrations within the endosome structures are not known, and they can notably vary even within specific microenvironments of the endosome (22, 23). However, the general trend is a decrease in sodium and calcium concentrations and an increase in potassium concentration during endosome maturation (24–26). Thus, changes in ion concentrations other than protons may well serve as a trigger for virus uncoating.

We showed previously that, at 37°C, albumin in combination with a neutral buffer mimicking the endosomal environment triggered the expansion of E1 without receptor engagement, creating an infectious A-particle (6). However, we did not show the individual effect of either albumin or ion changes to the overall conformational changes identified. Here, we show by real-time spectroscopy, gradient ultracentrifugation, cryo-electron microscopy (cryo-EM), and image reconstruction that endosomal ionic concentrations alone are sufficient to induce CVA9 expansion at a physiologically relevant temperature. Moreover, this treatment leads to unstable CVA9 virions which easily disintegrate, losing their genome. Albumin alone or in combination with endosomal ionic concentrations induces CVA9 expansion. We also show that particle expansion is a dynamic process yielding a mixed population of virions, as assessed by gradient ultracentrifugation and cryo-EM analysis. Using asymmetric flow field flow fractionation (AF4), we were able to physically separate expanded and intact particles and to confirm by mass spectrometry (MS) that expanded particles largely lack the small internal capsid protein VP4. Loss of VP4 and exposure of negatively charged amino acid residues in the capsid’s interior after expansion create a repulsive viral RNA-capsid interface that aids genome release.

## RESULTS

### faf-BSA or ions primed intact CVA9 to expanded virions.

**FIG 1:**
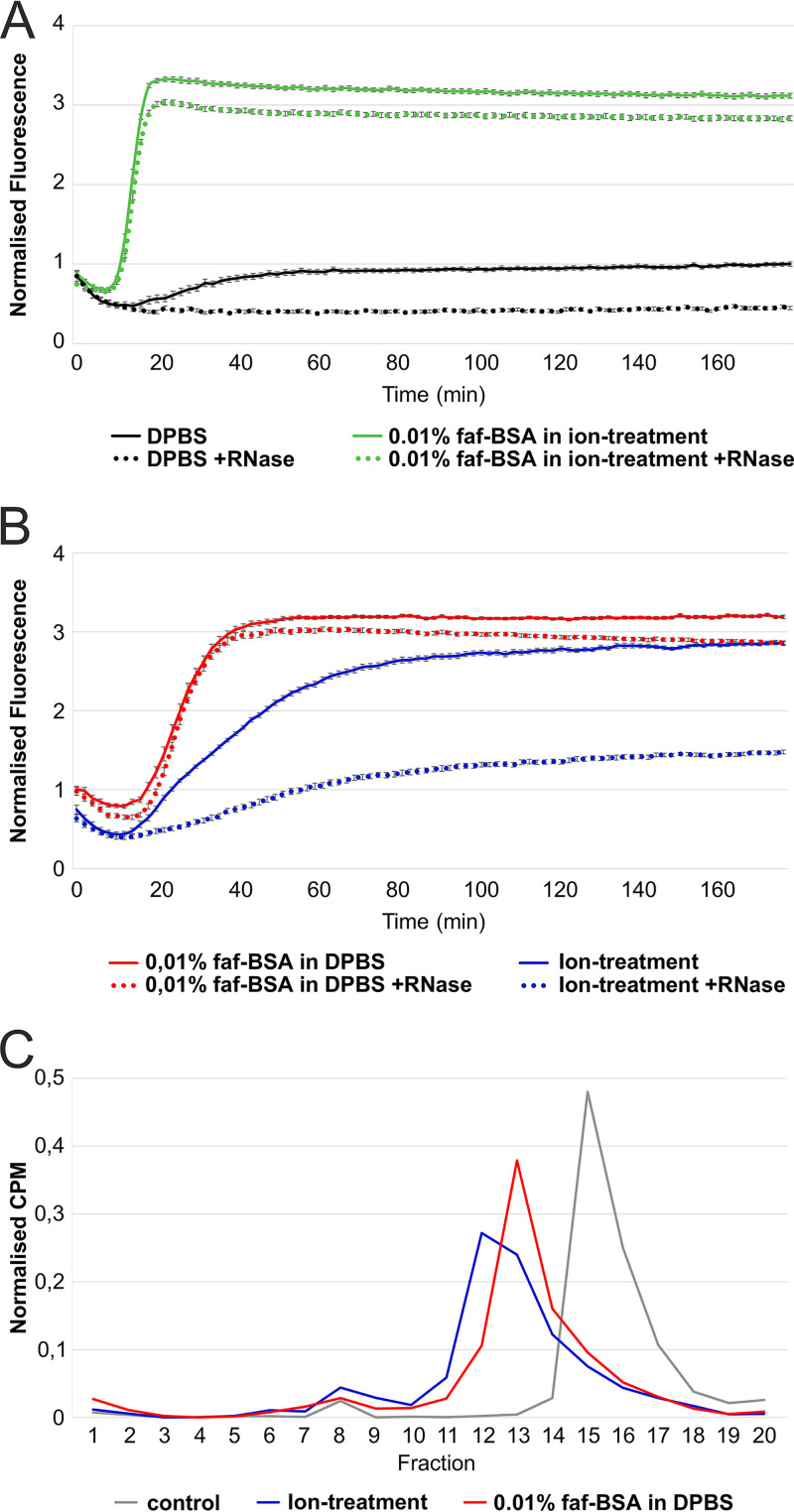
Endosomal ion and 0.01% faf-BSA treatments, individually or in combination, lead to CVA9 virion expansion. (A) Real-time spectroscopy measurements of the fluorescent dye SYBR green II accessibility to the CVA9 genome in the presence of 0.01% faf-BSA in an endosomal ionic environment (green) with (dotted line) or without (solid line) RNase. Untreated CVA9 in DPBS (black) with (dotted line) or without (solid line) RNase was used as a control. (B) Real-time spectroscopy measurements of fluorescent dye SYBR green II accessibility to CVA9 genome in the presence of 0.01% faf-BSA (red) or endosomal ion composition (blue) with (dotted line) or without (solid line) RNase. The fluorescence signal under the dotted curve indicates expanded virions, whereas fluorescence signal between solid and dotted curves originated from released genome, i.e., empty capsids. (C) Effect of 0.01% faf-BSA and endosomal ions on metabolically ^35^S-labeled CVA9 virions as analyzed by ultracentrifugation in a 5%-to-20% sucrose gradient and subsequent fractionation. CVA9 after endosomal ionic treatment (blue) and 0.01% faf-BSA treatment (red) is shown. Nontreated CVA9 in PBS-Mg buffer was used as a control (gray). Intact viruses were observed mainly in fractions 15 and 16, intermediate particles mainly in fractions 12 and 13, and empty particles in fractions 8 to 10. The exact buffer composition is given in Table 1.

We studied factors that lead to virion expansion or uncoating by monitoring changes in the viral RNA (vRNA) accessibility to the fluorescent dye SYBR green II using real-time spectroscopy. SYBR green II interacts with free nonencapsidated RNA, or it can access vRNA due to the pores in the expanded capsids (6, 27). In Dulbecco’s phosphate-buffered saline (DPBS), the virions were stable for 3 h according to the very low fluorescence readout throughout the measurement (Fig. 1A, black solid line; Table 1). Addition of RNase allowed us to distinguish between the signal originating from vRNA released from dissociated virions and the signal from expanded RNA-containing particles where the RNA is protected (Fig. 1A and B, dotted lines). For CVA9 in DPBS, the addition of RNase gave lower fluorescence values, indicating that either nucleic acids were present in the CVA9 preparation or vRNA was released from a small percentage of the virions and was degraded by the RNase (Fig. 1A, black dotted line). Incubation of CVA9 in 0.01% fatty acid-free bovine serum albumin (faf-BSA) induced a fluorescent signal starting at 20 min and reaching its plateau at 40 min (Fig. 1B, red solid line; Table 1). faf-BSA sequesters fatty acids from the virion. Addition of RNase in a parallel measurement showed only a slightly decreased fluorescence readout (Fig. 1B, red dotted line versus red solid line). This indicated that 0.01% faf-BSA in DPBS efficiently converted CVA9 into expanded particles and only a small fraction of virions dissociated, releasing their vRNA (Fig. 1B).

**TABLE 1:** Final buffer compositions used for treating CVA9 virions

Buffer	NaCl (mM)	Na_2_HPO_4_ (mM)	Total Na (mM)	KCl (mM)	KH_2_PO_4_ (mM)	K_2_HPO_4_ (mM)	Total K (mM)	MgCl_2_ (mM)	CaCl_2_ (mM)	faf-BSA (μM)	Palmitate (μM)
PBS-Mg (storage)	137	8	153	3	2		5	2			
DPBS	138	8.1	154.2	2.7	1.5		4.2	0.5	0.9		
Ion treatment	20		20		6	12	30	0.5	0.45		
Ion treatment + 15 μM palmitate	20		20		6	12	30	0.5	0.45		15
0.01 % faf-BSA^*a*^	138	8.1	154.2	2.7	1.5		4.2	0.5	0.9	1.52	
0.005 % faf-BSA	138	8.1	154.2	2.7	1.5		4.2	0.5	0.9	0.75	
0.0005 % faf-BSA	138	8.1	154.2	2.7	1.5		4.2	0.5	0.9	0.075	
0.005 % faf-BSA, + 15 μM palmitate	138	8.1	154.2	2.7	1.5		4.2	0.5	0.9	0.75	15
0.01 % faf-BSA in ion buffer (combined)	20		20		6	12	30	0.5	0.45	1.52	

a The 0.01% faf-BSA corresponds to a 20-fold excess of faf-BSA molecules per hydrophobic pocket in the virion (60 pockets per virus) when 10 μg/mL CVA9 was used, i.e., ~1.25 nM concentration of CVA9 with an estimated virion mass of 8 MDa.

The same method was applied to study the effect of the estimated endosomal ionic content on CVA9 virion expansion (Table 1). Ion treatment resulted in the fluorescent signal for vRNA being detected after 15 min, and this gradually increased, reaching a plateau in 3 h (Fig. 1B, blue solid line). The addition of RNase to the ion treatment reaction mixture reduced the fluorescent signal by half (Fig. 1B, blue dotted line versus blue solid line), indicating that during ion treatment a significant fraction of the CVA9 virions released their vRNA. Thus, incubation of CVA9 in the buffer mimicking endosomal content induced virion expansion and, in addition, RNA release from some virions.

The combined treatment caused an additive effect in the fluorescence signal kinetics, with expansion plateauing within 15 min (Fig. 1A, green lines). However, it considerably reduced RNA release from the virions compared to ion treatment alone (Fig. 1A, green dotted line, versus Fig. 1B, blue dotted line).

### Sucrose gradient analysis revealed sample heterogeneity.

Sucrose gradient analysis of metabolically labeled CVA9 confirmed the results of the fluorescence analysis. CVA9 treated with 0.01% faf-BSA in DPBS and separated by differential ultracentrifugation shifted the peak fraction compared to the control (Fig. 1C, red versus gray line). This shift indicated the formation of expanded particles, as their migration in the sucrose gradient was slower than that of the intact particles (28). In contrast, the ion treatment of CVA9 resulted in a slightly larger shift and a broader peak in the sucrose gradient (Fig. 1C, blue line) as well as a larger and broader peak of empty virions (fractions 8 to 10), in accordance with the fluorescence measurements (Fig. 1B). The overlapping peaks revealed incomplete separation of the particles in the gradient.

### AF4 allowed separation of expanded and intact particles.

**FIG 2:**
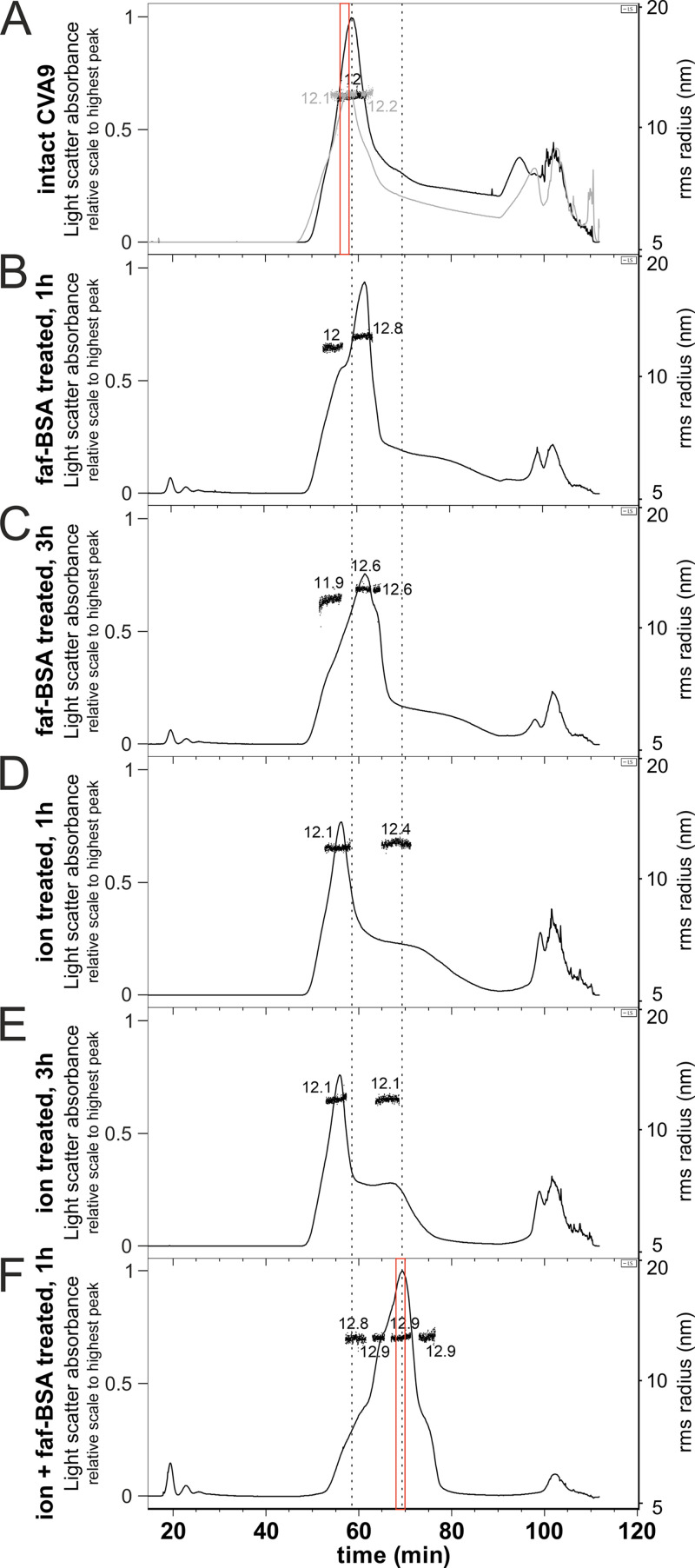
AF4 allows separation of the heterogeneous CVA9 virion population generated by 0.01% faf-BSA and endosomal ion treatments. (A) Two independent AF4 fractograms of untreated CVA9. (B) AF4 fractogram of CVA9 after 1-h treatment with 0.01% faf-BSA at 37°C. (C) AF4 fractogram of CVA9 after 3-h treatment with 0.01% faf-BSA at 37°C. (D) AF4 fractogram of CVA9 after 1-h treatment in endosomal ion environment at 37°C. (E) AF4 fractogram of CVA9 after 3-h treatment in endosomal ion environment at 37°C. (F) AF4 fractogram of CVA9 after combined treatment (0.01% faf-BSA in endosomal ionic environment) for 1 h at 37°C. In all panels, the *x* axis indicates time (1-mL fractions were collected at the rate of 0.5 mL/min); the *y* axis on the left shows light scattering (black or gray lines) and on the right the hydrodynamic radius is shown, as measured by online dynamic light scattering detector (black dots). The vertical dashed line on the left indicates the peak for intact particles and on the right the peak for expanded particles. AF4 fractions used for MS analysis are indicated by red boxes. The light scatter absorbance on all the fractograms was normalized to the highest light scatter absorbance peak, which is found in panel F.

AF4 has been shown to be useful in virus purification, compared to ultracentrifugation (29, 30), and in our experiments, AF4 allowed the physical separation of expanded particles (stabilized by faf-BSA) from intact ones and thus biochemical analysis of the proteins present (Fig. 2). Importantly, if VP4 is released from the expanded virions, it will flow through the 10-kDa-cutoff AF4 membrane used in this experimental setup and will not reach the fraction collector. Thus, the protein composition analysis of the virions separated by AF4 directly reflect only the protein composition in the particle.

The light-scattering signal in the AF4 fractograms of the untreated CVA9 showed a clear peak corresponding to particles with radii of gyration (Rg) of 12 and 12.1 nm (two separate measurements), giving geometric radii (R) of 15.5 and 15.6 nm, respectively (Fig. 2A; Table 2). The tail at 70- to 90-min elution indicated particle heterogeneity in the sample, and the peaks at the end of the fractogram (90 to 110 min) indicated aggregation. AF4 analysis of 0.01% faf-BSA-treated CVA9 revealed multimodal peaks, indicating a few transient states of the virions with lower diffusion coefficients compared to the intact virions (Fig. 2B and C). The Rg of particles corresponding to the highest peak were estimated at 12.8 nm (after 1-h treatment) and 12.6 nm (after 3-h treatment), giving R values of 16.5 and 16.3 nm, respectively, and indicating expanded virions. The peaks for soluble protein eluting at early time points (about 20 min) corresponded to BSA introduced in the buffer for the experiment.

**TABLE 2:** AF4 results^*a*^

CVA9 treatment	Rh (nm)	Rg (nm)	Rg/Rh	R (nm)
No treatment (control)	14.8	12.1	0.82	15.6
faf-BSA, 1 h	17.0	12.8	0.75	16.5
faf-BSA, 3 h	16.7	12.6	0.75	16.3
Ions, 1 h	14.7	12.1	0.82	15.6
Ions, 3 h	14.7	12.1	0.82	15.6
Combined treatment, 1 h	17.0	12.9	0.76	16.7

a Rh, hydrodynamic radius; Rg, radius of gyration; R, geometric radius. The geometric radius was estimated using the following equation: (Rg)^2^ = (3/5)R^2^.

Similar to the real-time spectroscopy measurements, ion-treated CVA9 showed slow virion conversion to expanded particles, as the main peak corresponded to the intact virions with an estimated Rg of 12.1 nm (R, 15.6 nm). The shoulder at the elution times 65 to 75 min corresponded to expanded virions. The Rg measurements on the shoulder were less reliable due to the lower signal and the extended elution time, indicating heterogeneity (Fig. 2D and E). The slight shift of the peak for intact virions could be explained by the changes in ion composition introduced by treatment (29).

Combined treatment with both ion and 0.01% faf-BSA resulted in a clearly shifted peak with a particle population Rg of 12.9 nm (R, 16.7 nm) (Fig. 2F). The first shoulder of the peak eluting at 60 to 63 min (Fig. 2F) could correspond to the main peak of the 0.01% faf-BSA-treated CVA9 (Fig. 2B and C). The second shoulder of the sample eluting at ~65 min could correspond to the right shoulder of the CVA9 treated for 3 h with 0.01% faf-BSA only (Fig. 2F, compared with Fig. 2C). In the sample with the combined treatment, as the major peak (53 to 77 min) had no tail and there was the smallest aggregation peak, we concluded the particles were expanded and stabilized by the treatment. Overall, AF4 enabled us to separate the expanded particles from the intact ones in two distinct peaks, which we then used for determination of the particle’s protein composition by MS.

### Mass spectrometry confirmed that most of VP4 is lost in expanded virions.

In order to investigate if VP4 is present or absent in the expanded virions, we performed MS analysis on AF4 peak fractions of the untreated CVA9 and CVA9 after combined treatment (indicated in red in Fig. 2A and F). The MS results indicated reduced spectral counts for VP4 in the treated versus the control CVA9 compared with the total counts for the other structural proteins (see Table S1 in the supplemental material). In the control sample, 8.8% of the spectral counts for the structural proteins corresponded to VP4 (13 spectra out of 134), which agreed well with the stoichiometry-based calculations from the virion amino acid composition, where VP4 accounted for 7.8% of the total. In the treated CVA9 sample, the spectra for VP4 reached only 0.9% (5 out of 533), indicating that most of the VP4 was lost in the expanded particles. Due to the fractionation in AF4, free VP4 was removed from the particle peak; thus, the MS analysis reflected only the protein present in the particles.

### Palmitate prevented particle expansion.

**FIG 3:**
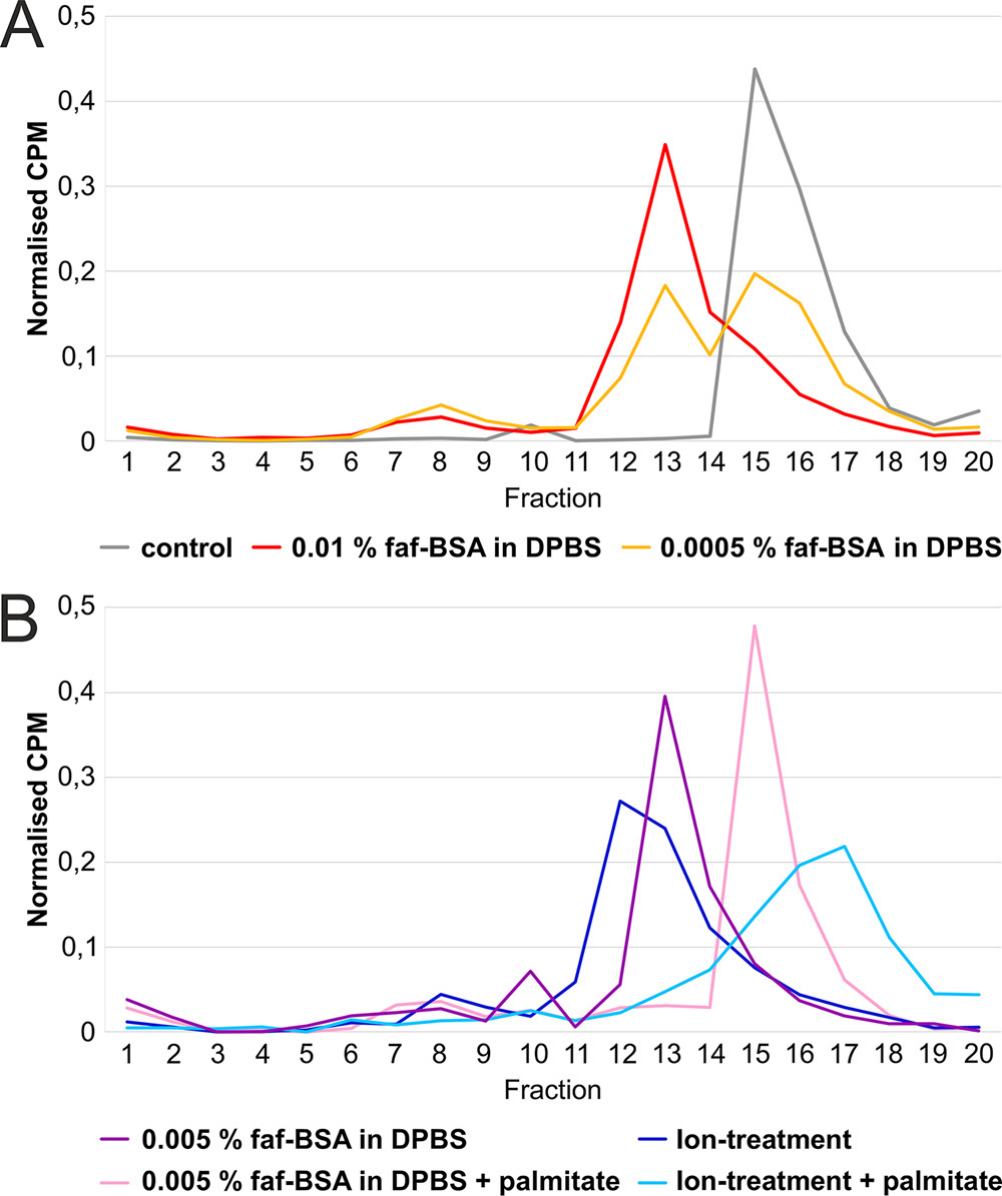
CVA9 expansion dependency on faf-BSA and palmitate concentrations. (A) Fractionation of untreated (gray), 0.01% faf-BSA-treated (red) or 0.0005% faf-BSA-treated (orange) CVA9 after separation in a 5%-to-20% sucrose gradient using ultracentrifugation. The peak for intact virions corresponded to fractions 15 to 17, for expanded virions to fractions 12 to 14, and for empty virions to fractions 7 to 10. (B) Analysis of CVA9 treated with 0.005% faf-BSA in the presence of an excess of palmitate (pink) after ultracentrifugation in a 5%-to-20% sucrose gradient. For comparison, 0.005% faf-BSA-treated CVA9 was ultracentrifuged the same way without addition of palmitate and is shown in purple. Similar analysis of CVA9 treated with endosomal ion concentrations with (blue) or without (light blue) addition of palmitate is also shown. The peak for intact virions corresponded to fractions 15 to 18, for expanded virions to fractions 12 to 14, and for empty capsids to fractions 7 to 10. Buffer composition is shown in Table 1.

The faf-BSA effect on CVA9 particle expansion is concentration dependent. When we treated the virus with 0.01% faf-BSA, equivalent to a 20-fold excess of faf-BSA molecules per hydrophobic pocket, we observed the efficient formation of expanded virions with only a small fraction corresponding to empty capsids, as analyzed by sucrose gradient differential centrifugation (Fig. 3A, red line versus gray line; Table 1). A 20-fold-lower concentration of faf-BSA (0.0005%), equal to a 1:1 molar ratio of faf-BSA per hydrophobic pocket, had only a moderate effect on the formation of expanded CVA9 particles (Fig. 3A, orange). Furthermore, in the case of the addition of an excess amount of palmitate (in relation to faf-BSA) to saturate both the faf-BSA and the virions, the CVA9 virions were protected from expansion (Fig. 3B, pink). Similar results were obtained when an excess amount of palmitate was added to the CVA9 ion treatment reaction mixture (Fig. 3B, blue versus light blue line).

### The heterogeneous particle population can be separated computationally.

**FIG 4:**
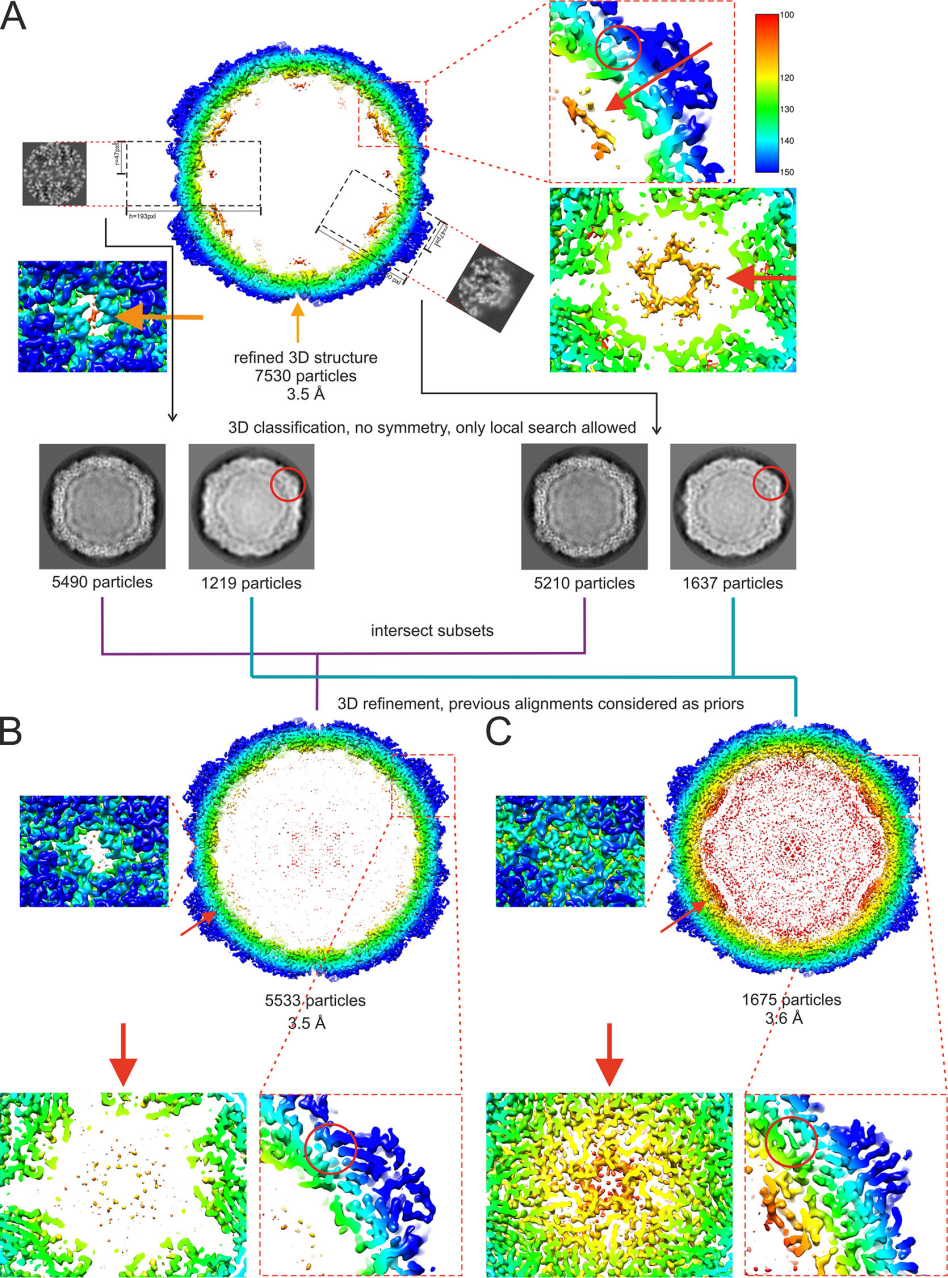
Workflow of focused classification applied to the faf-BSA-treated CVA9 data set to sort heterogeneity. (A) Cross-section of an initial faf-BSA-treated CVA9 reconstruction using standard 2D and 3D classifications. Features of interest at the 2-fold and 5-fold axes were enlarged, as well as the position of the hydrophobic pocket. Despite the nominal resolution at 3.5-Å resolution, the density under the 5-fold axis was much more ordered than expected. Focused classification on either the 2-fold or 5-fold axis yielded two distinct classes corresponding to intact and expanded particles, indicating that ordered density was due to contamination with intact virion particles in the reconstruction. The consensus classes were used for further refinement. (B) Cross-section of the faf-BSA-expanded CVA9 particle density map. Features at the 2-fold and 5-fold axes and the collapsed hydrophobic pocket are enlarged. (C) Cross-section of the native CVA9 particle density map obtained from a 2D class in the faf-BSA-treated CVA9 data set. In all three panels, features at the 2-fold, 5-fold, and the collapsed hydrophobic pockets are enlarged. Density maps are radially colored, in angstroms, from the particle center according to the color key on the right in panel A. Red circles indicate the position of VP1, where the hydrophobic pocket is occupied in the intact virion but collapsed in the expanded particle. Red arrows indicate representations where VP4 is organized in the virion but missing in the expanded particles.

**FIG 5:**
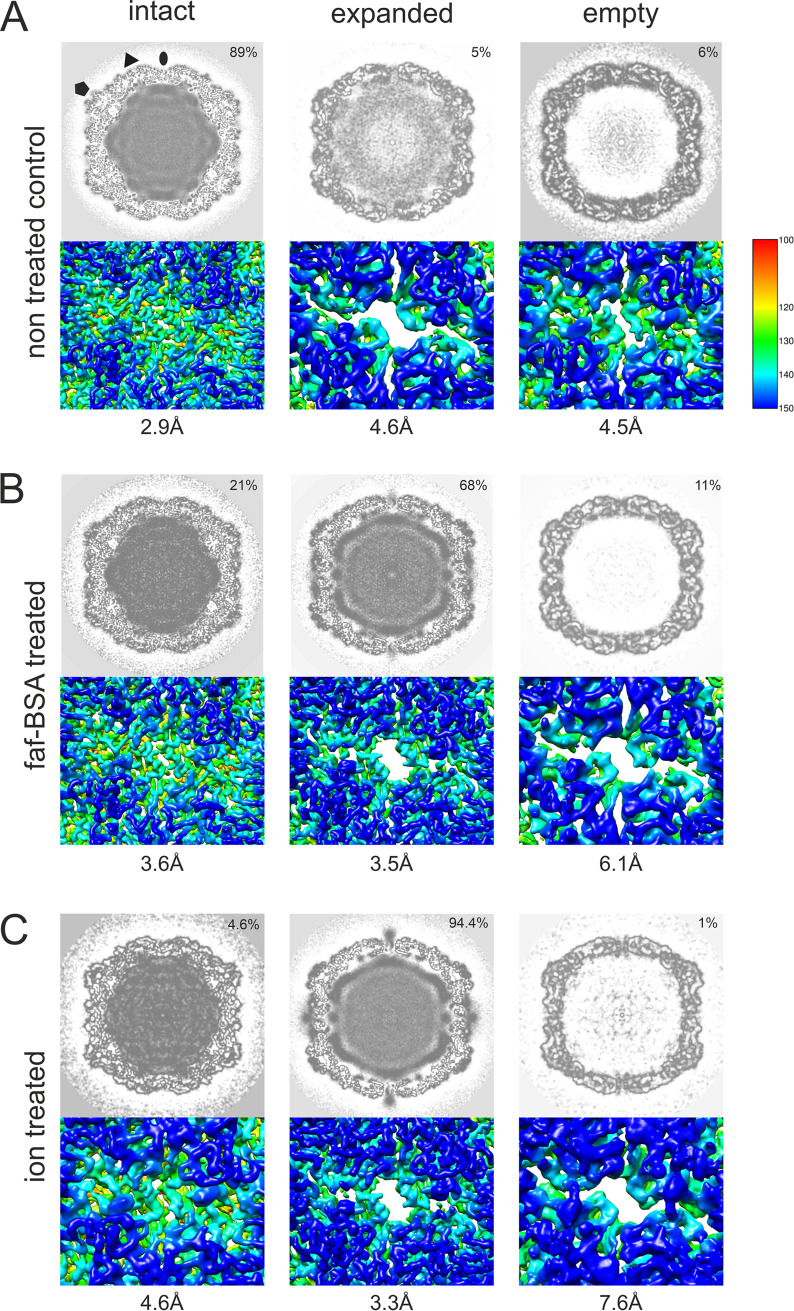
Heterogeneity of CVA9 virions in different data sets after cryo-EM and single-particle analysis. (A) Analysis of the data set of untreated CVA9 resulted in intact, empty nonexpanded, and expanded virions containing the genome. (B) Data set of 0.01% faf-BSA-treated CVA9 separated into expanded empty and filled capsids, in addition to intact particles. (C) CVA9 after treatment with endosomal ionic concentrations, in addition to main class of genome-containing expanded virions, yielded a small population of intact virions and expanded empty capsids. In all panels, top view shows central plane of the refined reconstructions, and below it is the enlarged view of a surface representation of a reconstruction, shown at the 2-fold axis and clearly indicating an opening seen in expanded virions. Distribution of particle classes is indicated on the top right corner, and resolution of the reconstruction is shown below the figure. Reconstructions are colored according to radial distance, in angstroms, from the particle center (color key shown on the left) and shown at 3 σ above the mean.

We used cryo-EM to analyze the intact CVA9 virions which were used as the input for faf-BSA or ion treatment, as well as the endpoint of the treatment achieved after 1 h (for faf-BSA treatment) or 3 h (for ion treatment) at 37°C. Multiple rounds of two-dimensional (2D) and 3D classifications, including focused classification, revealed several different particle types in all three data sets (Fig. 4 and 5; Table 3). The initial faf-BSA-treated CVA9 reconstruction showed strong density below the 5-fold vertices and below the openings on the 2-fold vertices (Fig. 4A). Further analysis using focused classification revealed that a contaminating class of nonexpanded particles contributed all of that density of interest to the reconstruction (Fig. 4B and C). This was a cautionary example of how the heterogeneity in the sample needs to be sorted out with complementary computational approaches (30). As expected, the main particle population in the control data set was that of intact particles (89%), but there were also expanded particles containing viral genome (5%) and empty nonexpanded capsids observed (6%) (Fig. 5A). The low number of expanded and empty particles in the control data set corresponded well with the low fluorescence signal from the untreated CVA9 sample (Fig. 1A, black lines); moreover, empty capsids may have released their RNA during storage, which was detected in the fluorescent measurement with RNase, or there was nucleic acid impurity in the sample (Fig. 1A, black dashed line). In faf-BSA- and ion-treated CVA9 data sets, the main populations, 68% and 94.4%, respectively, conformed to expanded virions containing the viral genome (Fig. 5B and C). In addition, the faf-BSA-treated CVA9 data set contained a significant number of intact (nonexpanded) and empty expanded particles, in agreement with the AF4 results showing a signal for intact particles in addition to expanded ones (Fig. 2B). The ion-treated CVA9 data set had the lowest percentage of intact and empty expanded particles (Fig. 5C). For free RNA, detected as a drop in fluorescence signal when RNase was added (Fig. 1B, blue dashed line), AF4 results showed a peak primarily for intact particles (Fig. 2D and E), and the overall low particle number picked from the micrographs (Table 3) indicated particle instability after ion treatment. In both the faf-BSA and ion-treated CVA9 data sets, the empty capsids were expanded with an enlarged capsid diameter and openings at the two-fold vertices (Fig. 5B and C).

**TABLE 3:** Particle heterogeneity

Type of particles	Native CVA9	BSA-treated CVA9	Ion-treated CVA9
Total no. of particles	21,365	8,061	9,055
Empty particles	1,275^*a*^	853^*b*^	93^*b*^
Expanded particles	1,024	5 533	8,540
Nonexpanded particles	19,066	1,675	422

a Nonexpanded particles.

b Expanded particles.

### Cryo-EM showed that faf-BSA or ion treatment of CVA9 led to A-particle formation.

**FIG 6:**
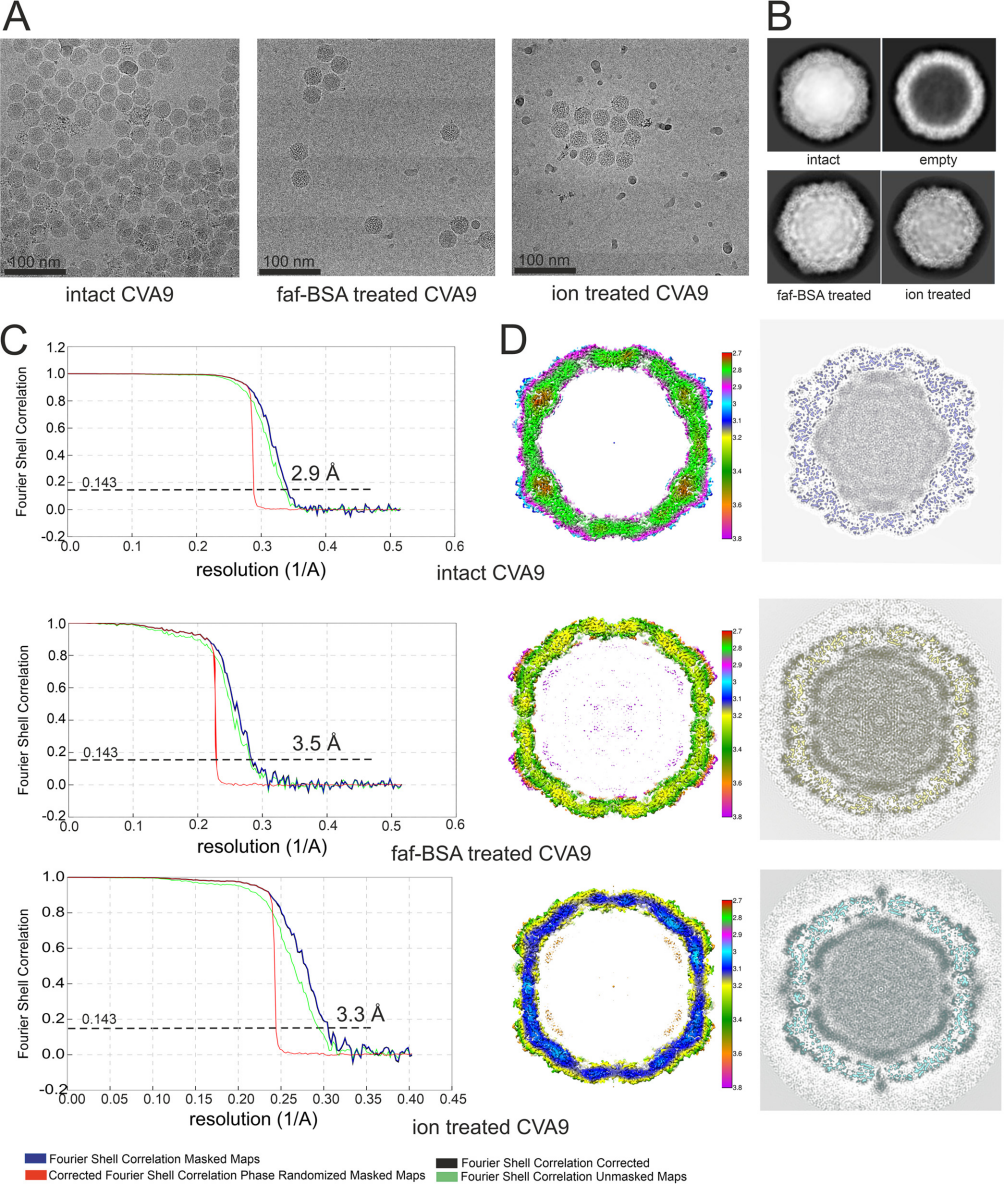
Cryo-EM data collection and single-particle analysis. (A) Representative micrographs from untreated, 0.01% faf-BSA-treated, and endosomal ionic conditions-treated CVA9 data sets. Scale bar, 100 nm. (B) Example 2D class averages obtained in Relion 2 during processing workflow for three data sets; the 2D class average corresponding to empty virions is from the untreated CVA9 data set. (C) FSC curves of final reconstructions for intact CVA9, 0.01% faf-BSA-treated and endosomal ion-treated CVA9, and estimated resolutions at a 0.143 threshold. (D, left) Central sections of the CVA9 reconstructions, colored according to local resolution in angstroms, as shown in the color keys. Reconstructions are shown at 2 σ above mean. (Right) Central planes of the reconstructions.

**FIG 7:**
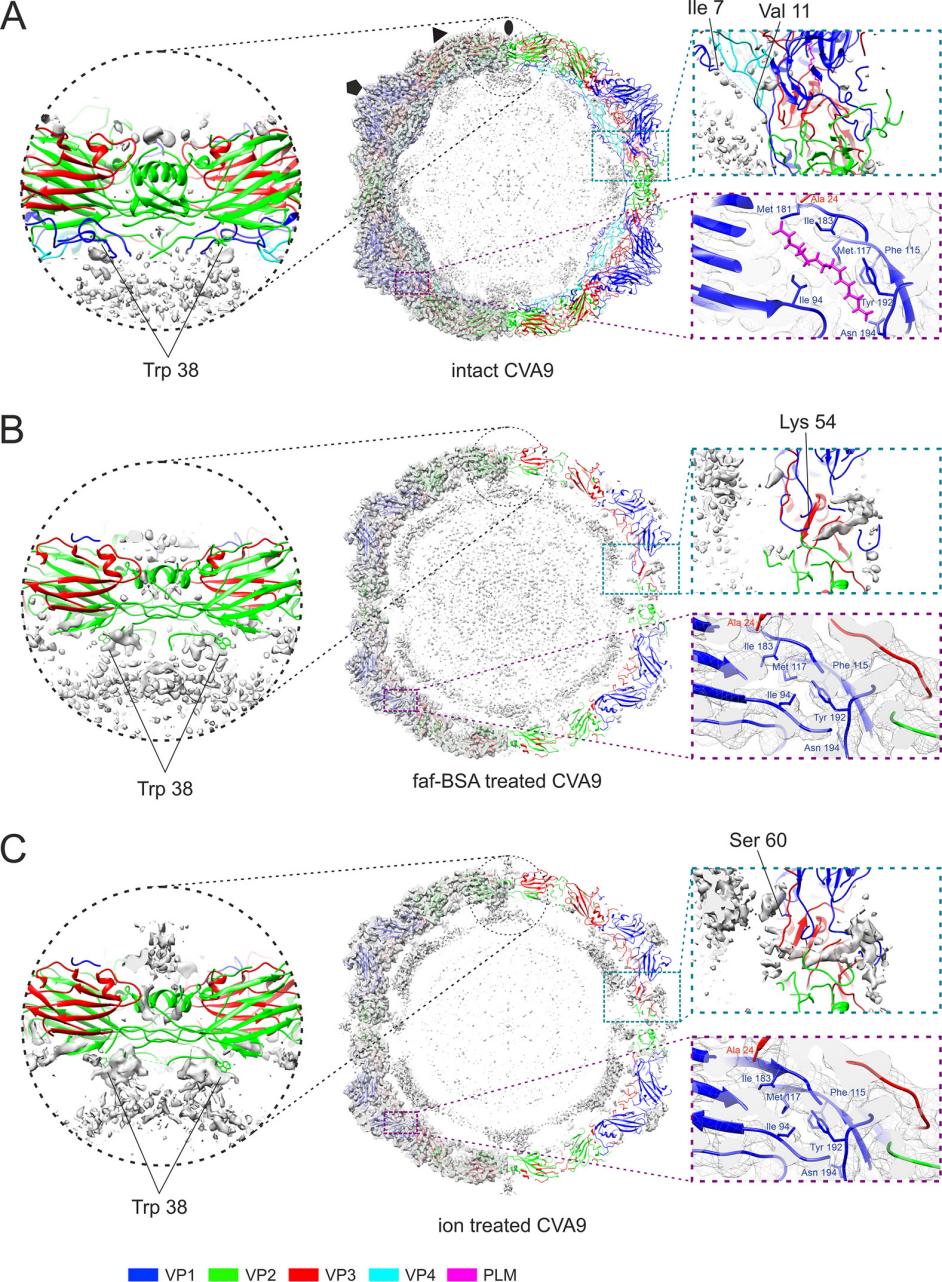
Comparison of reconstructions and atomic models of intact, 0.01% faf-BSA-treated, and endosomal ion-treated CVA9 virions. (A) Untreated CVA9 central section through the model fitted to the reconstruction shown at 3 σ above mean. (Left) Cryo-EM density with atomic model fit; (right) cryoEM density accounted for by the atomic model (not shown) to reveal the unmodeled density. Symmetr*y* axes are marked as follows: 5-fold, pentagon; 3-fold, triangle; 2-fold, ellipse. Enlarged view of the model at a 2-fold symmetr*y* axis highlights the vRNA contacts with Trp38 from VP2, observed in all three reconstructions. The enlarged view on the right shows that most of the density in the capsid region was accounted for. The VP1 N terminus extends on the inside of the capsid hugging VP4, and density for a lipid factor can accommodate an atomic model of a palmitate. (B) Central section through the atomic model fitted into the reconstruction of 0.01% faf-BSA-treated CVA9. On the right, half of the density that was accounted by an atomic model was removed to better visualize the unmodeled density. Enlarged view of vRNA contacts with Trp38 from VP2 is shown. View of unmodeled density near the VP1 N terminus (Lys54) spanning the capsid of expanded virions and collapsed hydrophobic pocket are zoomed on the right. (C) Central section through the atomic model fitted into the reconstruction of endosomal ion-treated CVA9. On the right, half of the density that was accounted by an atomic model was removed to better visualize the unmodeled density. Enlarged view of vRNA contacts with Trp38 from VP2 is shown, in which the density above 2-fold can also be seen. View of unmodeled density near the VP1 N terminus (Ser60) spanning the capsid of expanded virions and collapsed hydrophobic pocket are zoomed on the right.

To evaluate structural changes in the CVA9 virions induced by different treatments, we solved cryo-EM structures of the most populated classes from intact, faf-BSA-treated, and ion-treated CVA9 data sets to 2.9, 3.5, and 3.3 Å resolution, respectively (Table 4; Fig. 6). The well-defined density of the intact CVA9 virions showed features similar to those observed in capsids of other intact enteroviruses and accommodated well the model for all four structural proteins of CVA9 obtained by X-ray crystallography (PDB ID 1D4M) in a T=1 arrangement (31) (Fig. 6 and 7A). Similar to the X-ray structure, the intact virion reconstruction showed elongated density for a lipid factor placed in the hydrophobic pocket in VP1 (Fig. 7A), as well as a large portion of the myristoyl (MYR) group covalently attached to the VP4 N terminus. Furthermore, the base-stacking interaction between vRNA and VP2 Trp38 next to the 2-fold axis inside the intact virion could be clearly observed (Fig. 7A). The average radius of the intact virion, 15.6 nm, coincided well with the geometric radius measured in AF4 experiments (Table 2). Compared to the intact virions, the two cryo-EM reconstructions of CVA9 virions treated in two different ways, 0.01% faf-BSA or ion composition, yielded RNA-containing A-particles expanded by approximately 4% in diameter (Fig. 6 and Fig. 7, B and C). In addition, these reconstructions revealed more flexibility in capsid proteins and no density for the lipid factor. The MS results indicated that there were only nonstoichiometric amounts of VP4 present and no VP4 could be modeled into the reconstructions (Fig. 7; Table 4). The internal density seen in the difference map was attributed to vRNA (Fig. 7B and C).

**TABLE 4:** Cryo-EM data collection, refinement, and validation statistics

Category	CVA9	Ion-treated CVA9	BSA-treated CVA9
Data collection and processing			
Magnification	150,000	120,000	150,000
Voltage (kV)	200	200	200
Electron exposure (e^–^/Å^2^)	30	30	30
Defocus range settings (μm)	−0.8 to 1.8	−0.6 to 1.8	−0.8 to 1.8
Pixel size (Å)	0.97	1.24	0.97
Symmetry imposed	I2	I2	I2
Micrographs (no.)	2421	1,964	2,472
Initial particle images (no.)	28,818	9,702	15,179
Good particles	21,365	9,055	8,061
Final particle images (no.)	19,066	8,540	5,533
Map resolution (Å)	2.9	3.3	3.5
FSC threshold	0.143	0.143	0.143
Map resolution range (Å)	999–1.94	999–2.48	999–1.94
Refinement			
Map sharpening *B* factor (Å^2^)	−50	−70	−70
Model composition			
Nonhydrogen atoms			
Protein residues			
VP1	1–7, 11–283	60–127, 138–203, 206–283	54–127, 137–282
VP2	10–260	14–27, 29–43, 50–259	14–27, 30–45, 50–260
VP3	1–238	3–175, 185–233	3–175, 184–233
VP4	MYR-1–14, 23–68		
Ligands	Lipid factor		
RMSD^*a*^			
Bond lengths (Å)	0	0.88	0.87
Bond angles (°)	0.59	1.00	0.99
Validation			
MolProbity score	1.30	1.27	1.19
Clashscore	2.1	0	0
Poor rotamers (%)	0	2.05	1.64
Ramachandran plot			
Favored (%)	95.47	90.72	90.92
Allowed (%)	4.33	5.86	7.17
Disallowed (%)	0.2	3.42	1.91

a RMSD, root mean square deviation.

Further comparison of the two expanded particle reconstructions showed subtle differences between them (Fig. 7B and C). First, less of the VP1 N terminus was ordered in the ion-treated CVA9, where the first modeled residue was Ser60, compared to Lys54 in faf-BSA-treated CVA9 (Fig. 7). The order of the E1 VP1 N terminus after combined treatment was similar to that of faf-BSA-treated CVA9 (PDB ID 6O06) (6). In addition, external loops of VP1 were missing residues Asp128 to Asp136 residues in faf-BSA and Asp128 to Met137, Gln204, and Arg205 residues in ion-treated CVA9 models (Table 4). In VP2, residues Cys28, Ala29, and Glu46 to Ala49 in faf-BSA and Cys28, Asp44 to Ala49, and Ala260 in ion-treated CVA9 models were missing (Table 4). In VP3, surface-exposed residues Tyr176 to Glu183 in faf-BSA and Tyr176 to Tyr184 in ion-treated CVA9 models were missing (Table 4). Second, a poorly defined density was seen on the ion-treated, but not BSA-treated, surface above the 2-fold opening (Fig. 7B and C). The 3D classification of particles from ion-treated CVA9 refinement, which was focused on the 2-fold symmetr*y* axis, did not result in a better-defined density above the 2-fold opening, preventing a detailed atomic interpretation (Fig. 4). Altogether, cryo-EM revealed more flexibility in the ion-treated than in the faf-BSA-treated CVA9 capsid. This was in agreement with the fluorescence measurements that showed that ion treatment of CVA9 virions led to more unstable A-particles, which lost their vRNA and had become accessible to RNase (Fig. 1B, blue lines). In contrast, the faf-BSA treatment of CVA9 generated more stable A-particles, as seen by the fluorescence assay and AF4 (Fig. 1 and 2).

**FIG 8:**
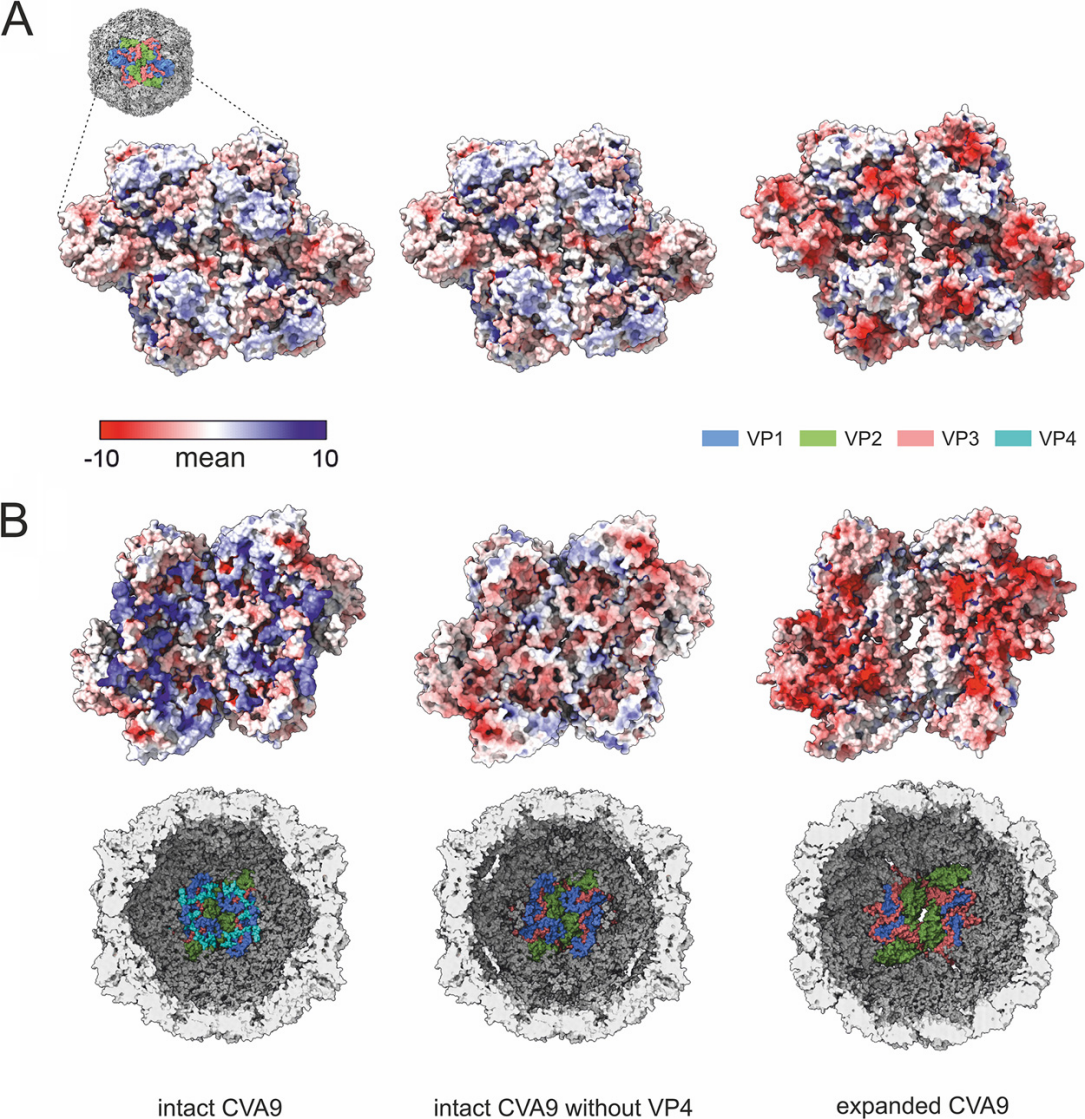
Calculations of electrostatic potentials show the significant change from a positive to a negative charge on the inner surface of the capsid when the intact virion (PDB ID 8AT5), the intact virion with VP4 removed computationally prior to calculating the electrostatic potential (PDB ID 8AT5), and the expanded virion (PDB ID 8AW6) are compared down a 2-fold axis of symmetry. (A) Electrostatic potentials are shown on the outer surface of the capsid for a cluster of four asymmetric units. The proteins contributing to the electrostatic potential are shown in the schematic diagram of the virion surface and are similar for all three renderings. (B) Electrostatic potentials are shown on the inner surface of the capsid for a cluster of four asymmetric units. The proteins contributing to the electrostatic potential are shown in the schematic diagrams of the virion inner surface. Electrostatic potentials are shown according to the color key in panel A. Blue, VP1; green VP2; salmon, VP3; cyan, VP4.

The average distribution of vRNA in the capsid changed with expansion (Fig. 6). In the intact virion, the RNA was tethered to the capsid via the VP2 Trp38, but it also was in close contact all over the capsid interior. Following faf-BSA or ion treatment, the average shape of the RNA expanded, reflecting the shape of the expanded capsid. However, apart from the tethering at Trp38, the intimate contact was lost (Fig. 6). Electrostatic potential calculations for the capsid proteins in intact and expanded virions indicated a significant shift to a much more negatively charged surface inside the expanded capsid generating repulsive vRNA-capsid interactions that could account for the observed changes and contribute to the vRNA exit (Fig. 8). To dissect the contribution of the loss of VP4, we also calculated the electrostatic potential of a nonbiological state, that of the intact virion shown without the contribution of VP4. From this image, it was evident that although VP4 did contribute significantly to the positive charge on the inside of the capsid, it was also the conformational changes of the other three capsid proteins during expansion that resulted in the much more highly negatively charged interior (Fig. 8).

## DISCUSSION

In enteroviruses E1 and E12 and coxsackievirus B3, serum albumin promotes virion expansion in a dose-dependent manner by sequestering the lipid factor from the VP1 hydrophobic pocket, as shown in *in vitro* experiments (6, 20, 21). Here, we studied and compared the roles of serum albumin and endosomal ion concentrations at neutral pH in triggering CVA9 expansion and genome release. We showed that when used independently, both albumin and endosomal ion concentrations caused particle expansion. However, the rate and extent of the order depended on the condition. The CVA9 treatment with albumin induced expansion in the majority of the particles exposing the VP1 N termini in about 30 min, whereas most of the particles still contained vRNA (Fig. 1B, 2, and 7B). The ion treatment led to a relatively slower particle expansion, driving the capsid to a more disordered state and the loss of vRNA from a significant population of particles (Fig. 1B, 2, and 7C; Table 4). Both effects could be prevented by an excess of palmitate preventing the collapse of the VP1 hydrophobic pocket, a necessary event for expansion (Fig. 3B). When virions were treated with faf-BSA, the lipid factor was bound more strongly by the albumin than by the capsid, causing the capsid to expand. In the case of ion treatment, lipid factor dissociation from the particle might have been driven by changes in counter ions so that the vRNA swelled, destabilizing the capsid for expansion.

A number of pieces of evidence indicate that the endosomes are the final location for picornavirus uncoating and vRNA release into the cytosol (32–35). Our experiments supported this idea by showing that changes in cation concentrations to those mimicking the endosomal environment slowly converted CVA9 into expanded virions. Moreover, under these conditions, a significant fraction of the virions lost their vRNA or dissociated completely, as shown by AF4 and real-time fluorescence measurements (Fig. 1 and 2). Viral RNA genomes inside the capsids are extremely compact, despite the repulsive negative charges of the RNA backbone phosphates (36). Tight vRNA folding depends on both the RNA sequence specificity and the presence of cations, which compensate for the repulsive interactions between negatively charged RNA backbones enabling close juxtaposition of two phosphate groups. RNA phosphate oxyanions have significant affinity to Mg^2+^ due to its size and charge density, which allows small interatomic distances in RNA-Mg^2+^ clusters, giving high stability (37). RNA association with Na^+^ and Ca^2+^ is not as stable, because of the lower charge of Na^+^ and the bigger size of Ca^2+^ ions. The most abundant cations that associate with highly folded RNAs are Mg^2+^ and Na^+^ (38). As mentioned before, during endosome maturation there is a tendency for a decrease in Na^+^ and Ca^2+^ ions and increase in K^+^ ions (22, 23). There is no information about changes in Mg^2+^ concentration in endosomes. Because of the changes in ion composition in endosomes, K^+^ ions might replace Na^+^ and perhaps Mg^2+^ ions in the compact vRNA, leading to conformational changes and expansion or swelling of the vRNA. Furthermore, the comparison of electrostatic potential on the internal surface of intact and expanded virions showed that vRNA-capsid interactions became repulsive in expanded virions due to conformational changes and the loss of much of the VP4, thus further fostering genome release (Fig. 8). In support of this, altered vRNA-capsid interactions in an A-particle of human rhinovirus 2 have been described (39).

In conclusion, the enterovirus capsid expansion required for release of the genome in the endosome can be triggered by albumin in the serum, but equally it can be triggered in a receptor- and acidic pH-independent manner by the environment of the endosomal lumen.

## MATERIALS AND METHODS

### Virus production and purification.

Green monkey kidney (GMK) cells were purchased from the American Type Culture Collection (ATCC) and were maintained in Eagle’s minimum essential medium supplemented with 10% fetal bovine serum (FBS), 1% GlutaMAX, and 1% antibiotic-antimycotic solution in a chamber environment adjusted to 37°C and 5% CO_2_. For CVA9 production, semiconfluent 5-layer bottles of GMK cells were infected with CVA9 (ATCC; Griggs strain, GenBank accession number D00627.1), using a multiplicity of infection of 3.8 in medium containing 1% FBS until total cell detachment (at 37°C with 5% CO_2_ for 20 to 24 h). The cells and medium were collected and freeze-thawed three times, and the cellular debris was centrifuged into a pellet (JA-10 rotor, 6,080 rpm, 30 min, 4°C). The virus-containing supernatant was mixed with polyethylene glycol 6000 (8% [wt/vol]; Sigma-Aldrich, St. Louis, MO, USA) and NaCl (2.2% [wt/vol]) at 4°C overnight. The precipitate was pelleted (JA-10 rotor, 8,000 rpm, 45 min, 4°C) and resuspended in R-buffer (10 mM Tris-HCl [pH 7.5], 200 mM MgCl_2_, 10% [wt/vol] glycerol). The resuspension was treated with 0.3% (wt/vol) sodium deoxylate (Sigma-Aldrich) and 0.6 (vol/vol) Nonidet P-40 (Sigma-Aldrich) for 30 min on ice and centrifuged (TX-200 rotor, 4,700 rpm, 15 min, 4°C) to pellet the remaining cellular membranes. The virus-containing supernatant was loaded on top of a linear 10-mL 5-to-20% sucrose gradient prepared in R-buffer and centrifuged (SW-41 rotor, 35,000 rpm, 2 h, 4°C). The gradient was collected into 500-μL fractions, and the virus-containing fractions were detected using a NanoDrop 1000 spectrophotometer (Thermo Scientific, Waltham, MA, USA). The virus-containing sucrose fractions were diluted at least 1:5 in 2 mM MgCl_2_-PBS, and the virus was pelleted by centrifugation (70Ti rotor, 35,000 rpm, 2 h, 4°C) and resuspended in 2 mM MgCl_2_-PBS. The concentration of the purified virus sample was measured using a NanoDrop instrument, and the stock was stored at −80°C in small aliquots.

### ^35^S-labeled virus production and purification.

The ^35^S-labeled virus was produced in semiconfluent 80-cm^2^ cell culture bottles. The bottles were infected with CVA9 (ATCC, Griggs strain) in low-methionine-cysteine medium supplemented with 1% FBS for 3 h, after which the culture medium was changed to a low-methionine-cysteine medium supplemented with 1% FBS containing 50 μCi/mL [^35^S]methionine-cysteine. After total cell detachment (20 to 24 h), the cells were collected and freeze-thawed three times. After pelleting the cellular residue (TX-200 rotor, 4,000 rpm, 15 min, 4°C), the supernatant was collected and Tween was added in a final concentration of 0.1%. Solution was incubated on ice for 30 min, centrifuged (TX-200, 4,700 rpm, 15 min, 4°C), and loaded on top of a 2-mL 40% sucrose cushion on ice. The cushion with the virus was centrifuged (SW-41, 30,000 rpm, 2.5 h, 4°C), after which liquid above the cushion and a 500-μL fraction from the cushion was collected to waste, the remaining three virus-containing 500-μL fractions were diluted into 2 mM MgCl_2_-PBS, and the virus was pelleted (70Ti, 35,000 rpm, 3 h, 4°C). The pellet was resuspended into 1.5 mL of 2 mM MgCl_2_-PBS and separated in 5 to 20% sucrose gradient in R-buffer (SW41, 35,000 rpm, 2 h, 4°C). After collecting 500-μL fractions, the virus was identified using a liquid scintillation analyzer TRI-carb 2910 TR (PerkinElmer, Waltham, MA, USA). The virus-containing fractions were collected and pelleted as described above. The pellet was dissolved in 2 mM PBS-MgCl_2_ and stored at −80°C in small aliquots.

### Analysis of treated CVA9 by ultracentrifugation in sucrose gradient.

One microgram of unlabeled, purified CVA9 was mixed with 1 to 3 μL of ^35^S-labeled CVA9 (demonstrating ~1,000 cpm), and faf-BSA (Sigma-Aldrich) or buffer, giving final concentrations as indicated in Table 1, was added to obtain a final volume of 100 μL. For molar ratio calculations between the pocket, faf-BSA, and palmitate (Sigma-Aldrich), an estimated molecular weight of 8 MDa was used for CVA9 and the weights provided by the supplier were used for faf-BSA and palmitate. Samples were incubated at 37°C for 1 h (faf-BSA treatment) or 3 h (ion treatment) before gradient separation. The control samples (purified CVA9 in storage buffer) (Table 1) were kept on ice during the incubations. After the incubation, the samples were pipetted on top of linear 5-to-20% sucrose gradients prepared in R-buffer and centrifuged (SW41, 35,000 rpm, 2 h, 4°C). After centrifugation, 500-μL fractions were collected and mixed with scintillation cocktail (Ultima Gold MW; PerkinElmer), and the radioactivity of each fraction was measured (Tri-Carb 2910 TR; PerkinElmer).

### AF4.

The AF4 experiments were performed using the Eclipse NEON FFF system (Wyatt Technology, Santa Barbara, USA) which included an Agilent 1260 Infinity II pump and autosampler, an analytical long channel with a dilution control module (Wyatt Technology), and an Agilent 1260 fraction collector. The AF4 device was coupled to an Optilab refractive index detector (Wyatt Technology), DAWN multiangle light scattering detector (MALS) with 18 angles (Wyatt Technology), online dynamic light scattering detector embedded in one of the DAWN angles (Wyatt Technology), and a UV detector (Agilent) set at 280 nm. Regenerated cellulose membrane at 10-kDa cutoff (Wyatt Technology lot number RIJB19432) and a Technology 525-μm spacer (Wyatt Technology lot number 247096) was used as the accumulation wall. Focusing was performed at a cross-flow velocity of 2 mL/min. Sample was injected at 0.2 mL/min. Elution was performed using constant cross-flow velocity of 3 mL/min for 80 min; channel flow was 1 mL/min; detector flow was 0.5 mL/min. The AF4 flows were controlled via VISION 3.1 software (Wyatt Technology). Channel temperature was set to 25°C. DPBS filtered through a 0.1 μm filter was used as the mobile phase for all experiments. Before sample runs, system setup was validated by running 30 μg of BSA solution (2 mg/mL). For all experiments, sample corresponding to 10 μg of purified CVA9 was injected. ASTRA 8.1 software was used for data analysis.

For AF4 analysis CVA9 was treated essentially the same way as for cryo-EM sample preparation. Briefly, 12 μg of purified CVA9 (0.8 μg/μL) was mixed with faf-BSA at a final concentration of 0.01% in DPBS, giving a 136-μL total volume. The sample was incubated for either 1 or 3 h at 37°C prior to injecting into the AF4 device. To analyze the effect of endosomal ionic content, purified CVA9 virions (12 μg) were incubated in ion treatment buffer (136 μL, total volume) for either 1 or 3 h at 37°C and then analyzed by AF4. Combined treatment was done by mixing purified CVA9 (12 μg) with 0.01% faf-BSA in ion treatment buffer in a 168-μL total volume and incubated for 1 h at 37°C, prior to AF4 analysis in a volume of 20 μL (for control), 140 μL (for ion treated and combined treatment), or 113 μL (faf-BSA treated).

Results obtained by MALS and light-scattering detectors were used to calculate the particle radius of gyration (Rg) and hydrodynamic radius (Rh), respectively. The Rg/Rh ratio was between 0.75 and 0.82, indicating that the shape of the particles present in different samples corresponded to a hard sphere, confirming the expected particle shape in the peak fractions (Table 2) (40, 41).

### Real-time fluorescence measurements.

Conversion of CVA9 virions to expanded and empty or dissociated particles, which release their RNA, was observed by real-time spectroscopy as described earlier (6, 27). In short, 1 μg of virus was mixed with buffers containing different ion and supplement (faf-BSA or palmitate) concentrations in the presence of 10× SYBR green with or without RNase A. Buffer details are provided in Table 1. The total volume of each reaction mixture was 100 μL. The increase in fluorescence was observed using a PerkinElmer 2030 multilabel reader Victor X4 (PerkinElmer) with F485 lamp filter and F535 emission filter for 3 h at 37°C, and the fluorescence levels with and without RNase were compared to estimate whether the virus was in an intact, expanded, or dissociated form. A blank measurement with all factors other than the virus was registered and subtracted from the corresponding values obtained during the measurements with virus-containing samples. The obtained fluorescence values were normalized against the DPBS measurement and reported in arbitrary units (AU).

### LC-MS analysis.

The peak fraction corresponding to the intact virions in the AF4 run of untreated CVA9 (Fig. 2A) and the peak fraction corresponding to the expanded virions in the AF4 run of CVA9 after combined 0.01% faf-BSA and ion treatment (Fig. 2F) were used for liquid chromatography (LC)-MS analysis. First, each fraction (1 mL in total) was heated at 60°C for 5 min to inactivate the virus. Then, 1 mL of lysis buffer (8 M urea buffer, 50 mM NH_4_HCO_3_, phosphatase inhibitors [Sigma-Aldrich catalog number P2745], and protease inhibitor cocktail [Sigma-Aldrich catalog number P8340]) was added to each fraction on ice. Undissolved particles were removed by centrifugation at 16,000 × *g* for 10 min at 4°C. Samples were reduced with Tris(2-carboxyethyl)phosphine (Sigma-Aldrich), and alkylated with iodoacetamide. Samples were diluted with 50 mM ammonium bicarbonate (Sigma-Aldrich catalog number 213-911-5) to reduce the urea concentration to <2 M. Sequencing-grade modified trypsin (Promega catalog number PRV5111) was added to the samples, and mixtures were incubated for 16 h at 37°C. Finally, the trypsin-digested samples were desalted with C_18_ BioPureSPN minicolumns (Nest Group catalog number HUM S18V). A detailed description of the method used here was previously published (42).

The desalted samples were analyzed using an EvoSep One liquid chromatography system coupled with a hybrid trapped ion mobility quadrupole time-of-flight mass spectrometer (Bruker timsTOF Pro2). The samples were directly loaded on Evotips and separated with an 8-cm by 150-μm analytical column (EV1109; Evosep) using the 60-samples/day method (21-min gradient time). Mobile phases A and B were 0.1% formic acid in water and 0.1% formic acid in acetonitrile, respectively. The MS analysis was performed in the positive-ion mode using data-dependent acquisition in online parallel accumulation-serial fragmentation (PASEF) mode (43) with 10 PASEF scans per topN acquisition cycle. Raw data (.d) from timsTOF Pro were searched using MSFragger (44) against the home-made CVA9 entries for the capsid proteins. The sequence of CVA9 Griggs isolate was used (45). Carbamidomethylation of cysteine residues was used as static modification. Amino-terminal acetylation and oxidation of methionine were used as the dynamic modifications. Trypsin was selected as the enzyme, and a maximum of two missed cleavages was allowed. Both instrument and label-free quantification parameters were left to default settings.

### Cryo-EM sample preparation and data collection.

To analyze the effect of faf-BSA on CVA9 virion structure, 3 μg of purified CVA9 (0.8 μg/μL) was mixed with faf-BSA at a final concentration of 0.01% in DPBS, giving a 34-μL total volume. The sample was incubated for 1 h at 37°C prior to plunging. To analyze the effect of endosomal ionic content on CVA9 virion structure, purified CVA9 virions (3 μg) were incubated in ion treatment buffer (34-μL total volume) for 3 h at 37°C. For cryo-EM sample preparation, 3.0 μL of the above-described samples (or nontreated CVA9 virions as a control) were applied to glow-discharged grids (Quantifoil R1.2/1.3 holey carbon on 300 copper mesh) that were manually blotted with filter paper and flash-frozen in liquid ethane using a homemade plunger. Data were acquired at the Biocenter Finland National Cryo-EM facility, by using a FEI TALOS Artica transmission electron microscope operated at 200 kV. Movies were collected with a Falcon III direct electron detector at a nominal magnification of ×150,000 (for the CVA9 control and the CVA9 treated with faf-BSA) or ×120,000 (for the CVA9 treated with the endosomal ionic composition), giving a pixel size of 0.97 or 1.24 Å per pixel, respectively. Each movie consisted of 30 frames with an exposure time of 1 s per frame. The total electron dose was approximately 30 electrons per Å^2^. Movies were corrected for beam-induced motion by aligning and averaging all frames with Motioncor2, implemented in Scipion version 2.0 (46, 47).

### Image processing.

The contrast transfer function of the averaged micrographs was estimated with CTFFind4 in Scipion version 2.0 (48, 49). Micrographs with astigmatism and diffraction were excluded from further processing. For particle picking, two-step Xmipp3 was used from Scipion version 1.2, followed by 2D classification in Relion 2 (50–52). Initial 3D models were generated using 2,000, 1,005, and 8,012 particles for the CVA9 control, CVA9 ion-expanded, and CVA9 BSA-expanded groups, respectively, utilizing a 3D initial model protocol in Relion 2 (51, 53, 54). Particles from the best 3D classes were further refined and postprocessed in Relion 2. After postprocessing, particles were subjected to contrast transfer function (CTF) refinement and Bayesian polishing using default parameters in Relion 3 (51, 53–56). Local resolution of the maps was calculated in Relion 3 at a 15-Å sampling rate and 25-Å resolution threshold for randomizing phases (55, 57). However, at this stage, inspection of a 7,530-particle reconstruction of the BSA-treated CVA9 data set showed strong but poorly defined density beneath the capsid near the 5-fold axis (Fig. 4). To try to resolve this 5-fold density, we performed focused classification at the 5-fold symmetr*y* axis (Fig. 4). For this, a cylindrical mask of 46-Å radius and 145-Å height was applied with a center shift below the 5-fold symmetry axis, and only a small angular search was allowed (50, 58). The focused 3D classification separated all particles into two well-defined classes. Classes were further refined and postprocessed in Relion 3 to resolve characteristic features of expanded or intact particles, while the density below the 5-fold axis seen in the initial faf-BSA-treated CVA9 reconstruction disappeared and was attributed to VP4 density from contaminating intact particles (Fig. 4). Approximately 18% of all particles in the initial BSA-treated data set were found to be nonexpanded intact virions (Table 3). Similar results were obtained by 3D classification of the same set of particles focused on a 2-fold symmetry axis by applying a cylinder mask of 45-Å radius and 187-Å height and shifting the center of the mask so that the capsid region at the 2-fold axis would be covered (Fig. 4). A similar workflow of focused 3D classification was also applied to ion-treated CVA9 and control data sets, but these efforts did not result in any other distinct classes other than the major class. Although this procedure seemed to be robust in separating the intact and expanded particles, it was still not sufficient. Therefore, additional rounds of inspection in UCSF Chimera and classification in both 2D and 3D were carried out, culminating in the identification of intact virions, nonexpanded empty particles, expanded virions, and expanded empty particles. The majority particle type for the control and the two treatments were then CTF refined and postprocessed in Relion 3.

The forward scatter (FSC) curve was calculated, and the resolution of the final reconstructions was determined according to the gold standard FSC of 0.143 threshold criterion (59). The image processing and refinement statistics for each data set are shown in Table 4, and the focused classification workflow is shown in Fig. 4.

### Modeling and analysis.

The CVA9 atomic model PDB ID 1D4M was fitted into the CVA9 control reconstruction using UCSF Chimera 1.15 and manually adjusted in Coot 0.9.2 (60, 61). The initial model for the CVA9 A-particle was a homology model calculated in I-Tasser using the expanded E1 particle (PDB ID 6O06) as a reference (6, 62). I-Tasser-derived models were fitted into the density maps of faf-BSA-treated and ion-treated CVA9 using UCSF Chimera 1.15 and were manually adjusted in Coot 0.9.2. Residues for which densities were not observed in the cryo-EM maps were deleted, as indicated in Table 4. All three optimized models were further refined against the cryo-EM map in the Molecular Dynamics Flexible Fitting (MDFF) program, operated together with the NAMD and VMD programs (63–65). A scale factor of 1 was used to weigh the contribution of the cryo-EM map to the overall potential function used in MDFF. Simulations included 10,000 steps of minimization and 100,000 steps of molecular dynamics under implicit solvent conditions with secondary structure restraints in place. The refined models were validated using the MolProbity server (66).

Comparison of the maps was done in UCSF Chimera 1.15 by implementing the “subtract maps” feature. The maps were calculated at the same resolution and threshold values. Unmodeled densities were identified by creating a density map around the atomic model with the “molmap” command and subtracting it from the corresponding reconstruction.

### Calculations of electrostatic potentials of capsid proteins.

Final atomic models of capsid proteins of intact, intact without VP4, and faf-BSA-treated CVA9 were used for calculations of electrostatic potential. PDB files were converted to PQR using the PDB2PQR online tool (https://server.poissonboltzmann.org/pdb2pqr) with PROPKA at pH 7.0 to assign protonation states with a CHARMM force field and leaving additional options as the default settings. Output results were used for Adaptive Poisson-Boltzmann Solver calculations with the Poisson-Boltzmann equation (67). Input parameters were left as the default settings. The output PQR file was used for visualization in ChimeraX 1.3 (68, 69). Surface representation of the input asymmetric subunit PDB file was colored based on the PQR file electrostatic potential values in a range from −10 to 10 from the mean.

### Data availability.

The atomic models and cryo-EM maps generated during the current study are available in the wwPDB repositories with accession numbers PDB ID 8AT5 (10.2210/pdb8AT5/pdb), EMD-15634 (CVA9 control), PDB ID 8AW6 (10.2210/pdb8AW6/pdb), EMD-15692 (faf-BSA-treated CVA9), PDB ID 8AXX (10.2210/pdb8AXX/pdb), and EMD-15706 (ion-treated CVA9).
